# Extending the viability of human precision-cut intestinal slice model for drug metabolism studies

**DOI:** 10.1007/s00204-022-03295-1

**Published:** 2022-04-15

**Authors:** C. Biel, O. Martinec, B. Sibering, K. van Summeren, A. M. A. Wessels, D. J. Touw, K. P. de Jong, V. E. de Meijer, K. N. Faber, J. P. ten Klooster, I. A. M. de Graaf, P. Olinga

**Affiliations:** 1 Department of Pharmaceutical Technology and Biopharmacy, Groningen Research Institute of Pharmacy, University of Groningen, Groningen, The Netherlands; 2 Department of Pharmacology and Toxicology, Faculty of Pharmacy in Hradec Kralove, Charles University, Prague, Czech Republic; 3 Department of Medical Biochemistry, Faculty of Medicine in Hradec Kralove, Charles University, Prague, Czech Republic; 4 Innovative Testing in Life Science & Chemistry Group, Utrecht University of Applied Science, Utrecht, The Netherlands; 5 Department of Clinical Pharmacy and Pharmacology, University Medical Center Groningen, Groningen, The Netherlands; 6 Department of Pharmaceutical Analysis, Groningen Research Institute of Pharmacy, University of Groningen, Groningen, The Netherlands; 7 Department of Hepato-Pancreato-Biliary Surgery and Liver Transplantation, University of Groningen, University Medical Centre Groningen, Groningen, The Netherlands; 8 Department of Gastroenterology and Hepatology, University Medical Centre Groningen, Groningen, The Netherlands; 9 Department of Pharmacokinetics, Toxicology & Targeting, Groningen Research Institute of Pharmacy University of Groningen, Groningen, The Netherlands

**Keywords:** Human precision-cut intestinal slices, Viability, Organoid medium, Ex vivo model, Drug metabolism

## Abstract

Human Precision-cut intestinal slices (hPCIS) are used to study intestinal physiology, pathophysiology, drug efficacy, toxicology, kinetics, and metabolism. However, the use of this ex vivo model is restricted to approximately a 24 h timeframe because of declining viability of the hPCIS during traditional culture. We hypothesized that we could extend the hPCIS viability by using organoid medium. Therefore, we cultured hPCIS for up to 72 h in organoid media [expansion medium (Emed) and differentiation medium (Dmed)]. After incubation, we assessed culture-induced changes on viability markers, specific cell type markers and we assessed the metabolic activity of enterocytes by measuring midazolam metabolite formation. We show that the adenosine triphosphate (ATP)/protein ratio of Emed-cultured hPCIS and morphology of both Emed- and Dmed-cultured hPCIS was improved compared to WME-cultured hPCIS. Emed-cultured hPCIS showed an increased expression of proliferation and stem cell markers, whereas Dmed-cultured hPCIS showed an increased expression of proliferation and enterocyte markers, along with increased midazolam metabolism. Using the Emed, the viability of hPCIS could be extended for up to 72 h, and proliferating stem cells remained preserved. Using Dmed, hPCS also remained viable for up to 72 h, and specifically rescued the metabolizing enterocytes during culture. In conclusion, by using two different organoid culture media, we could extend the hPCIS viability for up to 72 h of incubation and specifically steer stem cells or enterocytes towards their original function, metabolism, and proliferation, potentially allowing pharmacokinetic and toxicology studies beyond the 24 h timeframe.

**Supplementary Information:**

The online version contains supplementary material available at 10.1007/s00204-022-03295-1.

## Introduction

The intestine is one of the major organs where drugs are metabolized, transported and where drug-induced side effects appear (Niu et al. 2013; Scarpignato and Bjarnason 2019). Human models to study metabolism, toxicity, and transport of drugs are scarce, often complex, and expensive to use or are difficult to scale up for high throughput tests in the intestine (Dedhia et al. 2016). A major advantage of the precision-cut intestinal slices (PCIS) model is that the cellular and extracellular matrix composition of PCIS resembles the in vivo situation. Moreover, it is possible to relatively quickly scale up and prepare large amounts (hundreds) of slices from one piece of human tissue (M.M. Groothuis and A.M. de Graaf 2012).

It is known that there are species-specific differences in toxicology, metabolism, and transport of drugs (Martignoni et al. 2006b; Shanks et al. 2009). Using PCIS, it has been shown repeatedly that drug effects, metabolite formation, and transport of drugs differ when tissues of animals and humans are used (Martignoni et al. 2006a; Khan et al. 2009b, a). Considering the 3R’s of animal experimentation, reduction, refinement, and replacement, and the better translational potential of human tissue, the use of human PCIS (hPCIS) is preferred. Since the expression and function of important enzymes, transporters, and transcription factors are similar to the in vivo human levels in short-term incubations, hPCIS can be used to predict in vivo metabolism and transport of drugs (Khan et al. 2009b). However, when using hPCIS for longer incubations, for example, to study drug-drug interactions or induction, hPCIS do not retain their expression and function of CYP-enzymes and drug-transporters (Kerkhof et al. 2008; Martinec et al. 2021).

hPCIS are prepared from the mucosa of human intestine and thus contain, among others, the epithelial and crypt cells of the intestine (Graaf et al. 2010). A disadvantage of hPCIS is that the metabolic activity and general viability decrease over time. In a recent study, the gene expression changes of hPCIS (and slices of other organs and species) during culturing were described and showed a general pro-inflammatory response upon culture (Bigaeva et al. 2019b). In mouse and rat PCIS, culture-induced changes in cellular heterogeneity and deterioration of the PCIS viability during culture were described (Biel et al. 2020). Although it is known that also hPCIS viability decreases during standard culture, detailed characterization of the cellular composition and function during long-term hPCIS culture remains was not yet reported.

Previous studies have shown that by using organoid media components, some features of culture-induced changes in mouse and rat PCIS may be improved (Bigaeva et al. 2019a; Biel et al. 2020). Similar organoid media components have been used to grow both mouse and human intestinal organoids and epithelial cultures (Sato et al. 2011; Kozuka et al. 2017). We, therefore, hypothesized that hPCIS viability can be improved by using organoid media, instead of the standard culture medium that does not contain the growth factors and small molecules stimulating cell proliferation and differentiation that are present in the organoid medium.

Therefore, the aim of this study was to improve the viability of hPCIS and to steer specifically towards proliferating stem cells and metabolically active enterocytes in hPCIS during culture by using tailor-made organoid media (Table 1). We have chosen to investigate two different medium compositions both developed by the Research Centre for Healthy and Sustainable Living, Innovative Testing in Life Sciences and Chemistry at the Applied University Utrecht. Expansion medium (Emed), which was developed to promote stem cell proliferation and inhibit differentiation of these cells, and Differentiation medium (Dmed), developed to maintain the proliferating cells, but at the same time promote differentiation towards enterocytes in intestinal organoids (Table 1). To determine whether the organoid media could improve the viability of hPCIS during culture, changes in viability markers, such as adenosine triphosphate (ATP)/protein ratio, morphology, inflammation, and cell death were assessed. Furthermore, changes in cellular heterogeneity and function were determined by performing gene and protein expression analyses of specific cell type markers and metabolic activity of enterocytes by measuring midazolam metabolite formation. This study further enhances the knowledge of the changes in the hPCIS epithelium during culture. Furthermore, we show that by adding specific supplements to the culture medium, hPCIS tissue viability but also specific crypt and epithelial cell viability can be extended for up to 72 h. Therefore, this study contributes to the development, characterization, and use of hPCIS for future toxicology, pharmacokinetic and pharmacological studies.

**Table 1:** Media compositions

Component	Supplier	Final concentration	WME	DMEM/F12	Emed	Dmed
WME medium	Gibco™, Life Technologies, Bleiswijk, The Netherlands		x			
DMEM/F12	Gibco™, Life Technologies, Bleiswijk, The Netherlands			x	x	x
Glucose	Merck, Darmstadt, Germany	25 mM	x			
P/S	Sigma-Aldrich, Saint Louis, USA	100 U/mL			x	x
Gentamycin	Gibco™, Life Technologies, Bleiswijk, The Netherlands	50 μg/mL	x	x	x	x
Amphotericin B	Gibco™, Life Technologies, Bleiswijk, The Netherlands	2.5 μg/mL	x	x	x	x
Pyruvate	Sigma-Aldrich, Saint Louis, USA	1 mM			x	x
HEPES	Sigma-Aldrich, Saint Louis, USA	10 mM			x	x
FCS	Sigma-Aldrich, Saint Louis, USA	10% (v/v)			x	x
R-spondin 1		10% (v/v)			x	x
WNT3a		10% (v/v)			x	x
DMH1	Sigma-Aldrich, Saint Louis, USA	1.3 mM			x	x
CHIR99021	Sigma-Aldrich, Saint Louis, USA	4.3 mM			x	
Y27632	Selleckchem, Munich, Germany	10 mM			x	
Valproic acid	Sigma-Aldrich, Saint Louis, USA	1 mM			x	

## Materials and methods

### Human intestine

Healthy intestinal tissue for research was obtained with approval from the Medical Ethical Committee of the University Medical Centre Groningen. The tissue originated from the resected intestine (i.e., proximal jejunum) of patients who underwent a pylorus-preserving pancreaticoduodenectomy (PPPD) for a variety of indications (Table 2). After resection, the intestine was immediately stored in ice-cold Krebs–Henseleit buffer (KHB) and processed directly after collection.

**Table 2:** Patient characteristics

Patient No.	Gender	Age
1	F	23
2	M	66
3	F	70
4	F	64
5	F	55
6	M	56
7	F	63
8	M	67
9	F	81
10	M	77
11	M	74
12	F	68
13	F	71
14	F	63

### Preparation of human precision-cut intestinal slices

The tissue processing and slicing procedure was performed as previously described (Graaf et al. 2010). In brief, the intestine was cut open and flushed with KHB. The submucosa, muscularis, and serosa tissue were removed and the mucosa was cut into sheets. The sheets were embedded in 3% (w/v) agarose in 0.9% NaCl. Slices were prepared in ice-cold KHB by using the Krumdieck tissue slicer (Alabama Research and Development, Munford, USA). The hPCIS weighed 3 mg (≈ 400 µm thickness) and were stored in ice-cold KHB until the start of the experiment.

### Incubation of hPCIS

hPCIS were incubated in 12-wells plates in 1.3 mL Williams Medium E (Gibco™, Life Technologies, Bleiswijk, The Netherlands, Cat. no. 32551) (supplemented with 25 mM Glucose (Merck, Darmstadt, Germany), 50 µg/mL Gentamycin (Merck, Darmstadt, Germany) and 2.5 µg/mL Amphotericin B (Merck, Darmstadt, Germany), or in organoid media prepared from supplemented Dulbecco’s Modified Eagle’s Medium/Nutrient Mixture F12 (DMEM/F12) medium (Gibco™, Life Technologies, Bleiswijk, The Netherlands, cat. no. 15290026) (Expansion medium, Emed, and Differentiation medium, Dmed, Table 1), kindly provided by Utrecht University of Applied Science (Table 1). The supplements R-spondin-1 and Wnt3a-conditioned media were prepared as previously described (Bigaeva et al. 2019a). The slices were incubated for 24 h, 48 h, or 72 h at 37 °C, 80% O_2,_ and 5% CO_2_ while shaking horizontally (90 rpm) and the medium was changed every 24 h.

### Viability

As a measure of general viability, the ATP/protein content of hPCIS was determined according to a standard protocol (Graaf et al. 2010; Bigaeva et al. 2019a). In short, directly after slicing, and after incubation, slices were snap-frozen individually (*n* = 3 slices/ condition) in 500 µL sonication solution (2 mM EDTA in 70% Ethanol) and stored at − 80 °C until further use. The samples were thawed, homogenized, and centrifuged and ATP content was determined by using the ATP bioluminescence kit (Roche Diagnostics, Manheim, Germany) according to the manufacturer's protocol. The ATP content was normalized against the protein content, which was determined by Lowry assay (Bio-Rad DC Protein Assay, Veenendaal, The Netherlands).

### Gene expression

**Fig. 1:**
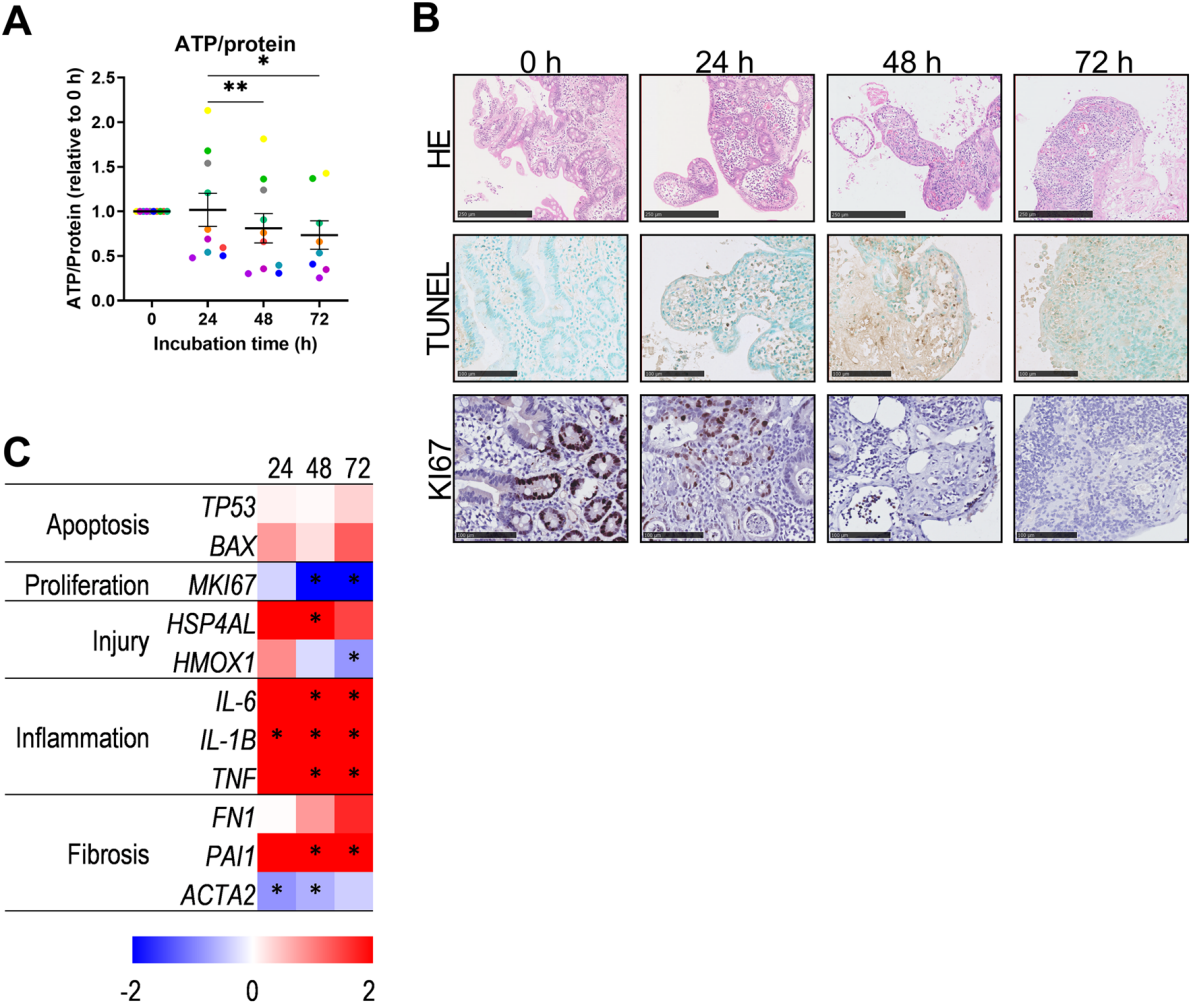
General viability of hPCIS incubated in the standard culture medium WME. hPCIS were incubated in WME for 24, 48, or 72 h. Relative ATP/protein ratios were determined (**A**). Dots represent the relative ATP/protein ratio of slices from individual donors the horizontal lines indicate mean ± SEM. Each color represents an individual donor. Representative photomicrographs of H&E, TUNEL, and KI67 stained sections of hPCIS (**B**). Photos were taken at 40× magnification, bars indicate 250 μm for H&E stained sections and 100 μm for TUNEL and KI67 stained sections. Heatmap of apoptosis, proliferation, injury, inflammation and, fibrosis gene expression relative to 0 h (**C**). The range indicates log2 normalized expression compared to 0 h, *n* = 5–7. *Indicate statistical difference compared to 0 h of incubation. One-way ANOVA or REML followed by Dunnett’s post-hoc test was performed to calculate statistical differences (*p < 0.05, **p < 0.01)

**Fig. 2:**
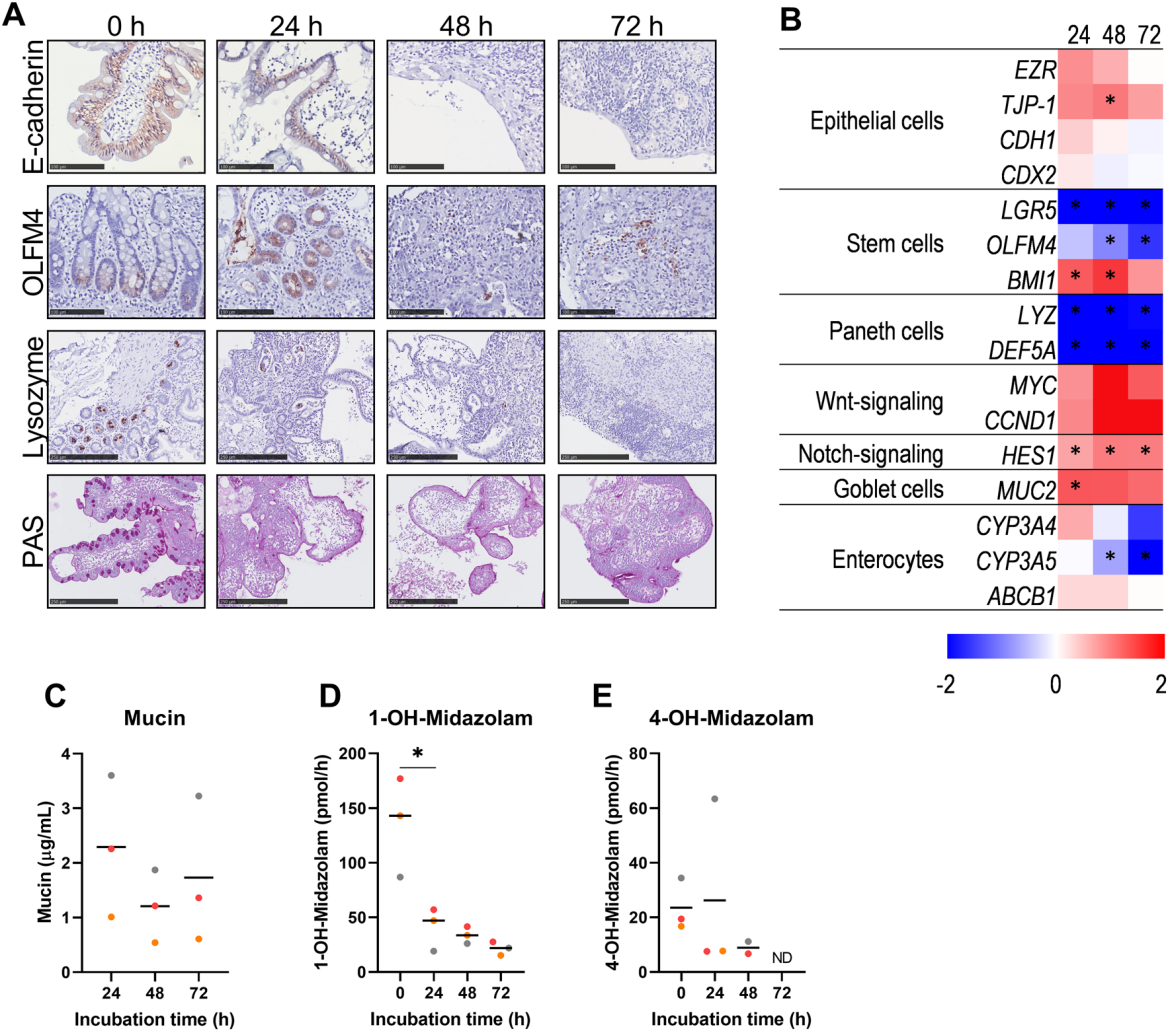
Cell types and metabolism of hPCIS incubated in WME. hPCIS were incubated in WME for 24, 48, or 72 h. Representative photomicrographs of E-cadherin, OLFM4, Lysozyme, and PAS stained sections of hPCIS (**A**). Photos were taken at 40× magnification, bars indicate 100 μm for E-cadherin and OLFM4 stained sections, and 250 μm for Lysozyme and PAS-stained sections. Heatmap of epithelial cell, stem cell, paneth cell, Wnt-signaling, goblet cell, and enterocyte gene expression relative to 0 h. The range indicates log2 normalized expression compared to 0 h, *n* = 5–7 (**B**). Mucin released in the medium over 24 h was determined using ELLA (**C**). Dots indicate mucin concentration in the culture medium (μg/mL), horizontal line indicates the mean. Directly after slicing, or in the last 3 h of incubation hPCIS were exposed to Midazolam and metabolite formation into 1-OH-midazolam (**D**), and 4-OH-midazolam (**E**) was measured in the medium, horizontal lines indicate the mean. Dots indicate mucin concentration (µg/mL) or metabolite formation rate (pmol/h) from hPCIS from individual donors. One-way ANOVA or REML followed by Dunnett’s post-hoc test was performed to calculate statistical differences compared to 0 h of incubation (*p < 0.05). *ND* not detected

**Fig. 3:**
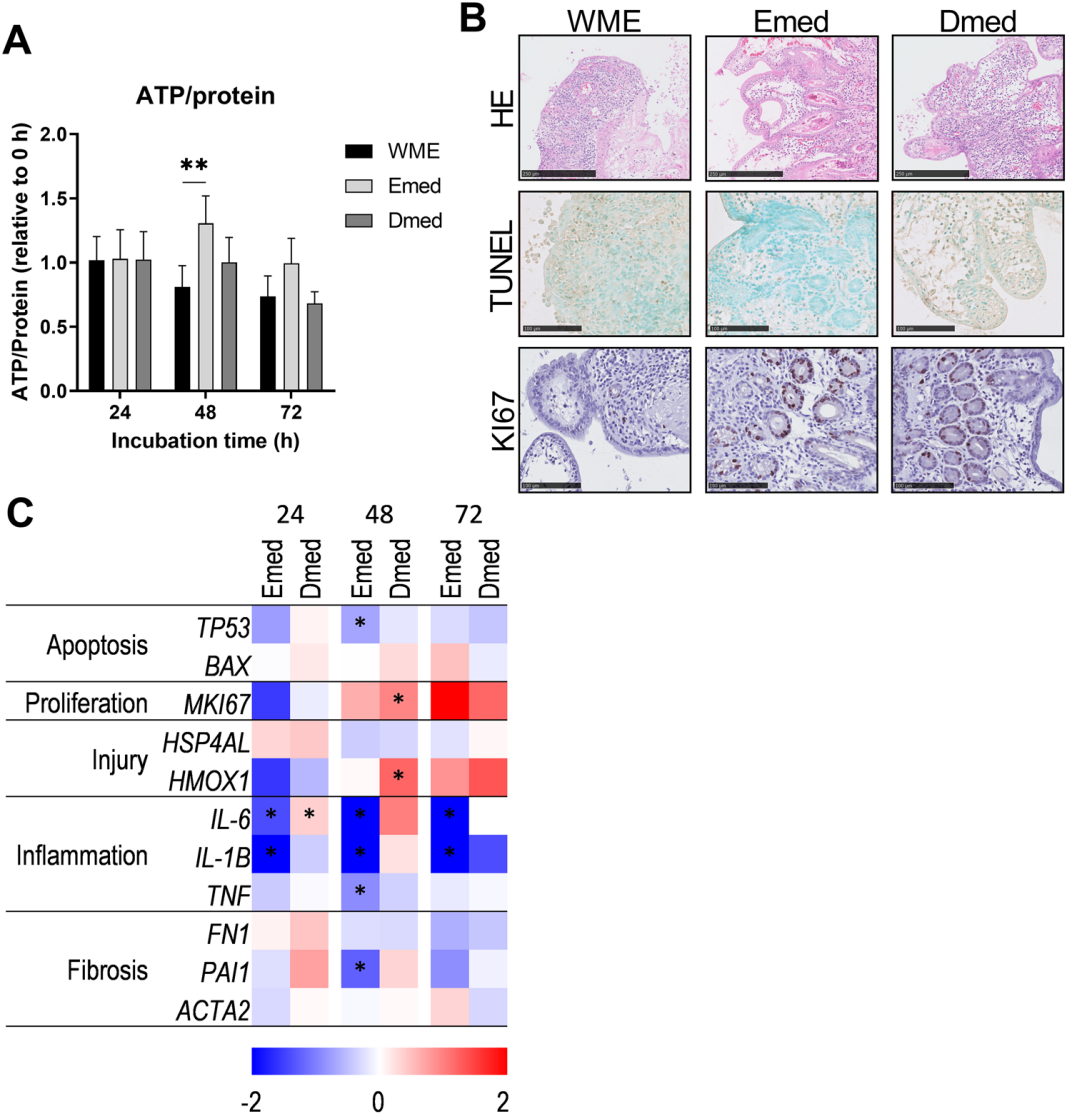
General viability of hPCIS incubated in different culture media. hPCIS were incubated in WME, Emed, or Dmed for 24, 48, or 72 h. Relative ATP/protein ratios were determined (**A**). Bars indicate mean + SEM, *n* = 7–9. Representative photomicrographs of H&E, TUNEL, and KI67 stained sections of hPCIS incubated for 72 h (**B**). Photos were taken at 40× magnification, bars indicate 250 μm for H&E stained sections and 100 μm for TUNEL and KI67 stained sections. Heatmap of apoptosis, proliferation, injury, inflammation, and fibrosis gene expression relative to WME cultured hPCIS of the respective time point. The range indicates log2 normalized expression compared to WME, *n* = 5–7 (**C**). REML followed by Dunnett’s post-hoc test was performed to calculate statistical differences compared to 0 h of incubation (*p < 0.05, **p < 0.01)

**Fig. 4:**
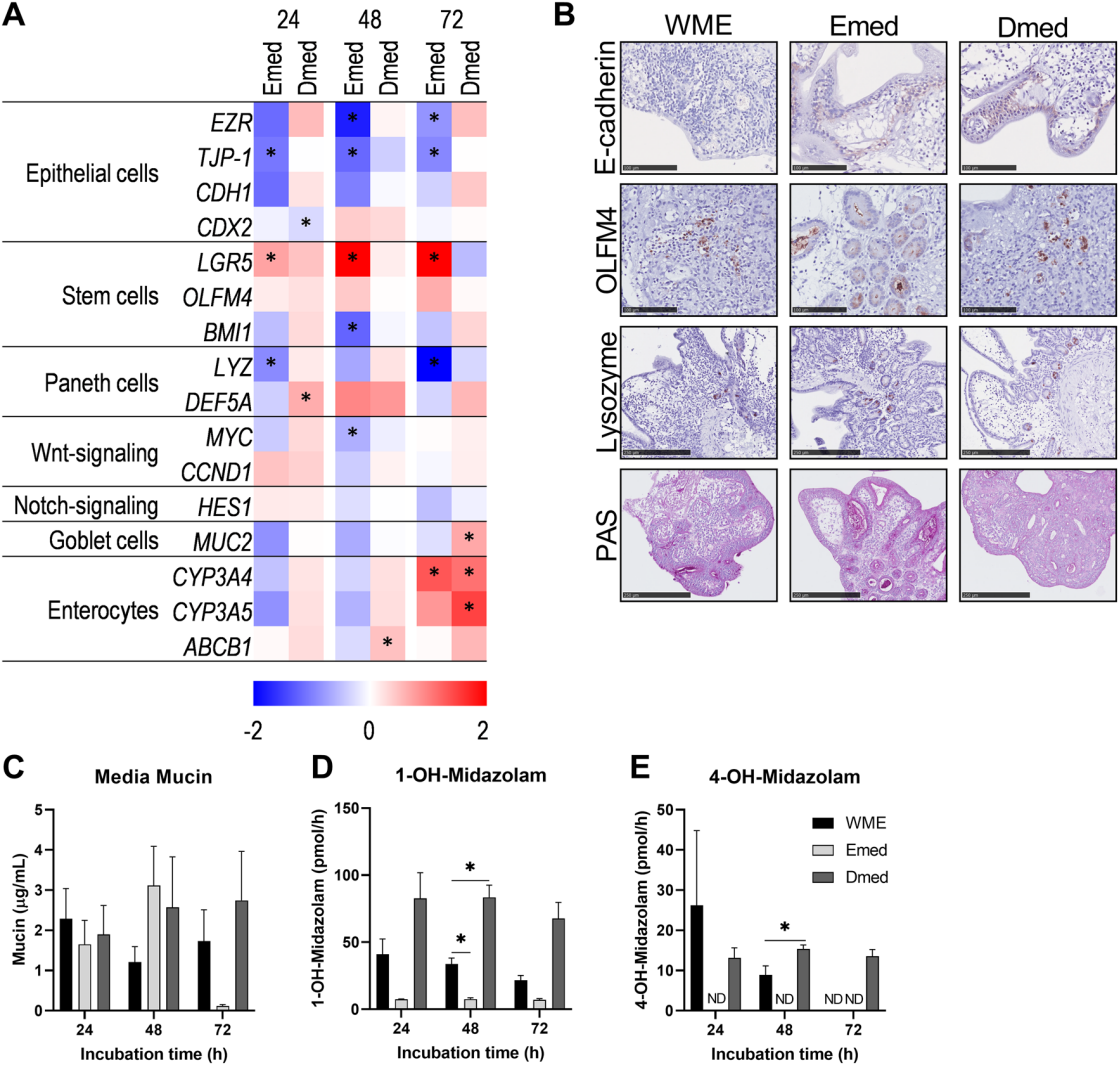
Cell type changes and function of hPCIS incubated in WME, Emed, or Dmed. hPCIS were incubated in WME, Emed, or Dmed for 24, 48, or 72 h. Heatmap of epithelial cell, stem cell, paneth cell, Wnt-signaling, goblet cells, and enterocyte gene expression relative to WME incubated hPCIS of the respective time point. The range indicates log2 normalized expression compared to WME, *n* = 5–7 (**A**). Representative photomicrographs of E-cadherin, OLFM4, Lysozyme, and PAS-stained sections of 72 h WME, Emed, or Dmed-incubated hPCIS (**B**). Photos were taken at 40× magnification, bars indicate 100 μm for OLFM4 and E-cadherin stained sections, and 250 μm for Lysozyme and PAS stained sections. Mucin released in the medium over 24 h was determined using ELLA (**C**). Bars show mucin concentration in culture medium (μg/mL) + SEM, *n* = 3. In the last 3 h of incubation, hPCIS were exposed to Midazolam, and metabolite formation rate into 1-OH-midazolam (**D**), and 4-OH-midazolam (**E**) was determined. Bars show metabolite formation rate (pmol/h) + SEM, *n* = 3. REML (RT-qPCR) or two-way ANOVA followed by Dunnett’s post-hoc test was performed to calculate statistical differences compared to 0 h of incubation (*p < 0.05). *ND* Not detected

To assess the effect of incubation of hPCIS in WME, Emed, and Dmed on gene expression of specific injury and cell type markers, RT-qPCR was performed. After slicing and incubation, pooled slices (*n* = 6 slices/condition) were snap-frozen and stored at − 80 °C until RNA isolation. RNA was isolated by using the FavorPrep tissue total RNA mini kit (Favorgen, Vienna, Austria) according to the manufacturer’s protocol. RNA content was measured by using Nanodrop (NanoDrop Technologies, Wilmington, USA) and cDNA was synthesized by using the Promega Reverse Transcriptase System kit (Promega, Leiden, The Netherlands) according to the manufacturer’s protocol. Gene expression of several general viability and cell type markers was determined by using 10 ng cDNA/reaction, primers (supplementary table 1), and the SYBR Green Real-Time PCR kit in the ViiA™ 7 Real-Time PCR system (Applied Biosystems) according to standard protocol. Expression levels were normalized against the housekeeping gene *RPLP0* and to the control (ΔΔCt method). Data is represented as 2^^−ΔΔCt^. Heatmaps shown in the figures represent the log2 normalized expression compared to 0 h (Figs. 1 and 2) or compared to WME (Figs. 3 and 4).

### Staining and immunohistochemistry

To determine the effect of incubation in WME, DMEM/F12, Emed, or Dmed medium on the morphology, cell death, and specific expression of cell type makers in hPCIS, several stainings were performed. Directly after slicing, and after incubation, slices (*n* = 3 slices/condition) were fixed in 4% buffered formalin for at least 24 h at 4 °C, dehydrated, embedded in paraffin, and sectioned in 3 µm sections. Morphological changes due to culture and medium use were visualized by Hematoxylin–Eosin (HE) staining according to a standard protocol. Maintenance of mucus-producing cells (goblet cells) was visualized by Periodic acid-Schiff (PAS) staining according to standard protocol. Terminal deoxynucleotidyl transferase dUTP nick end labeling (TUNEL) assay (TUNEL Assay Kit—HRP-DAB (ab206386), Abcam, Cambridge, UK) was used according to the manufacturer’s protocol.

Immunohistochemistry (IHC) for an epithelial cell marker (E-cadherin), a stem cell marker (OLFM4), a paneth cell marker (lysozyme), and a proliferation marker (KI67) was performed. Before the staining procedure, sections were deparaffinized and rehydrated. Heat-mediated antigen retrieval was performed in TRIS–EDTA buffer, pH 9 for 15 min in the microwave. Sections were blocked for 10 min by using PBS/2%BSA/2% rat serum. Primary antibody incubation was done for 1 h with 1:200 dilution for E-cadherin (Cell Signaling Technology PA, Leiden, the Netherlands, cat No. 3195S), 1:200 dilution for OLFM4 (Cell Signaling Technology PA, Leiden, the Netherlands, cat. No. 14369), 1:1000 dilution for Lysozyme (Proteintech, Manchester, UK cat. No. 15013-1-AP), and 1:1000 dilution for KI67 (Abcam, Cambridge, UK, cat. No. ab15580). The endogenous activity of peroxidases was blocked by 15 min incubation in 0.1% H_2_O_2_ in PBS. Goat anti Rabbit polyclonal antibody (1:50, 30 min, DAKO, Glostrub, Denmark cat. No. P0448) was used as the secondary antibody. Sections were stained with ImmPACT Nova RED (Vector Laboratories, Burlingame, USA) and counterstained with Hematoxylin.

Images were taken by using the Nanozoomer Digital Pathology Scanner (NDP Scan U10074-01, Hamamatsu Photonics K.K., Japan).

### Enzyme-linked lectin assay

To determine the mucus production of cultured hPCIS, Enzyme-linked lectin assay (ELLA) was performed as previously described (Biel et al. 2020). In short, medium samples were collected after each time point of incubation and stored at − 20 °C until measurement. Before the measurement, wells were coated for 2 h with wheat germ agglutinin (4 μg/mL in PBS) (Sigma-Aldrich, Saint Louis, USA, cat. No. 101845324), washed, and blocked with TBST. Samples and standard curve (0.08–10 μg/mL, Porcine Mucin type II, Sigma, 84082-64-4) were added and incubated for 1 h at 37 °C. For detection, samples were incubated with horseradish peroxidase-labeled soybean agglutinin (2 μg/mL in PBS, Sigma-Aldrich, Saint Louis, USA, cat. No. L2650) for 1 h at 37 °C. After washing, color development was done by 5 min incubation with 3,3′,5,5′-tetramethylbenzidine substrate (Sigma-Aldrich, Saint Louis, USA, cat. No. 1002623873), and the reaction was stopped using 1 M H_2_SO_4_. Absorbance at 450 nm was measured using a BMG FLuoStar-Omega. Signal-to-background ratio was calculated using a culture medium control.

### UHPLC-MS/MS midazolam metabolites

To assess the metabolic activity of CYP3A4 and CYP3A5 enzymes present in enterocytes, hPCIS were exposed to midazolam (MIDA), and MIDA and metabolites in the medium were measured. Directly after slicing, or 3 h before sampling, MIDA dissolved in methanol was added to the culture medium (~ 3000 µg/L). MIDA was kindly provided by the department of clinical pharmacy and pharmacology (University Medical Centre Groningen, Groningen, The Netherlands). After 3 h of MIDA exposure, 1 mL medium (per hPCIS), was collected and stored at − 80 °C until analysis.

For the quantification of midazolam and its primary metabolites 1-Hydroxymidazolam (1-OH-MIDA), 4-Hydroxymidazolam (4-OH-MIDA), in medium samples, an ultra-high performance liquid chromatography-tandem mass spectrometry (UHPLC-MS/MS) analytical method validated according to FDA and EMEA guidelines, was used (Wessels et al. 2021).

### Statistics

Experiments were performed with at least three slices per donor (technical replicates) from a total of three to ten donors (biological replicates). Statistical analyses were performed using Graph pad Prism 8.2 (GraphPad Software Inc., CA, USA). One-way ANOVA or Mixed effect model (REML) (when data points were missing) followed by Dunnett’s post-hoc test was used to compare the effect of incubation time during standard incubation in WME. Two-way ANOVA or Mixed effect model (REML) (when data points were missing) followed by Dunnett’s post-hoc tests were used to compare the different culture media to WME. A *p* value < 0.05 was considered statistically significant.

## Results

### hPCIS general viability and epithelial and crypt cell types deteriorate during culture in WME

To investigate whether hPCIS resemble the in vivo mucosal layer prior to, and during culture, ATP/protein determination, several stainings, and RT-qPCR for specific cell type markers and MIDA metabolism determination were performed in hPCIS collected prior to and during culture. During incubation, the relative ATP/protein ratio decreased gradually during an incubation time up to 72 h (Fig. 1A). HE staining showed that the epithelial layer is mostly intact directly after the slicing procedure, however, some signs of edema and in some cases, epithelial lifting was present (Fig. 1B). The morphological appearance of the hPCIS deteriorated during incubation. In the first 24 h of incubation, villi disappeared and crypts became disorganized. After 48 h and 72 h of incubation, the epithelium had completely flattened and crypts disappeared completely (Fig. 1B). As an indicator of cell death, TUNEL staining, specific for DNA-strand breaks were performed. Before culture, TUNEL staining was only present in a few cells, while TUNEL stained cells increased during incubation (Fig. 1B). Gene expression of apoptosis-related genes did not significantly change during incubation (Fig. 1C and S1). KI67 staining, specific for proliferating cells, showed abundant positive staining in the proliferating crypts before culture, while KI67 staining was not present in WME-cultured hPCIS after 48 and 72 h of incubation (Fig. 1B). This is in line with the gene expression of proliferation marker *MKI67* that was significantly decreased after 48 and 72 h of incubation (Fig. 1C and S1). Injury marker *HSP4AL* was significantly upregulated after 48 h of incubation. At the same time, WME-cultured hPCIS significantly increased gene expression of inflammation markers *Interleukin (IL)-6*, *IL-1B*, and *TNF* and fibrosis marker *PAI1* during incubation (Fig. 1C and S1). These results are in line with previous reports (Pham et al. 2015), indicating that the general viability of hPCIS decreased during incubation in WME medium, as indicated by the deterioration of ATP/protein ratio, hPCIS morphology, cell death, proliferation, and specific injury markers.

To investigate which cell types are the most affected during hPCIS culture, gene and protein expression of specific cell type markers were determined, and MIDA metabolism was assessed. The presence of epithelial cells (lateral E-cadherin staining) and stem cells (intracellular OLFM4 staining) and Paneth cells (intracellular Lysozyme staining) in the crypts were established by IHC staining (Fig. 2A). After 24 h incubation, E-cadherin translocation indicated the loss of polarization of epithelial cells. Epithelial, stem, Paneth, and goblet cells were absent after 48 h of incubation, as shown by the lack of E-cadherin, intracellular OLFM4, Lysozyme, and PAS staining (Fig. 2A). Interestingly, the gene expression of epithelial markers *EZR*, *TJP-1*, *CDH1,* and *CDX2* hardly changed during incubation (Fig. 2B and S1), in contrast to markers for specific cell populations. The gene expression of stem cell markers *LGR5* and *OLFM4* significantly decreased during incubation while *BMI1* increased in the first 48 h of incubation. Paneth cell markers *LYZ* and *DEF5A* were significantly decreased after 24 h of incubation (Fig. 2B and S2). Before culture, mucin-containing goblet cells were detected using PAS staining (Fig. 2A). Mucin-containing goblet cells were not detected in PAS-stained 24 h incubated hPCIS, while *MUC2* expression was increased at 24 h of incubation (Fig. 2A and B). Mucin excretion per 24 h by, among others, goblet cells, measured using ELLA, did not significantly change over 72 h of incubation (Fig. 2C). Enterocytes are the main epithelial cells responsible for the transport via, among others ATP-binding cassette protein (ABCB1) and metabolism of exogenous compounds, typically involving cytochrome P450 enzymes (CYPs). The mRNA expression of *ABCB1* hardly changed during 72 h of hPCIS incubation. To assess the metabolic capacity of the hPCIS enterocytes, hPCIS were exposed to MIDA for 3 h whereafter MIDA-metabolite formation rates (1-OH-MIDA and 4-OH-MIDA) were determined. Of note, neither midazolam nor the solvent had significant effects on the hPCIS relative ATP/protein ratio (Fig. S2). Directly after slicing, the hPCIS were able to metabolize MIDA into the primary metabolites 1-OH-MIDA and 4-OH-MIDA (formation rates: 135.6 ± 26.2 pmol/h and 23.5 ± 5.5 pmol/h, respectively, Fig. 2D & E), indicating active CYP3A4/5 enzymes. During incubation of hPCIS, *CYP3A4* expression did not significantly change while *CYP3A5* expression was significantly decreased after 48 and 72 h of incubation (Fig. 2B), This coincided by a reduced MIDA metabolism, as shown by decreased 1-OH-MIDA formation rates after 3 h MIDA exposure at 24 h (41.1 ± 11.4 pmol/h), 48 h (33.7 ± 4.5 pmol/h), and 72 h (21.6 ± 3.6 pmol/h) of hPCIS incubation compared to 0 h of incubation (135.6 ± 26.3 pmol/h), although this was only significant at the 24 h timepoint (Fig. 2D). 4-OH-MIDA formation after 3 h of MIDA exposure to hPCIS also decreased (ns) throughout the incubation and was not detectable in the medium of hPCIS incubated for 48 h in one donor and after 72 h for all donors (Fig. 2E). In conclusion, the presence and function of villi, as well as crypt located cells in hPCIS, diminish during the course of 72 h of incubation in the standard medium.

Downstream targets of two important intestinal stem cell signaling pathways (Wnt and Notch) are dysregulated during hPCIS incubation (Fig. 2B and S1). Wnt-downstream target, *LGR5* is downregulated during incubation. While *MYC* and *CCND,* targets of both Wnt-signaling, are increased (ns). Notch downstream target *HES1* is upregulated.

### Dmed and Emed improve hPCIS long-term viability

To extend the viability of ex vivo*-*cultured tissue, we cultured the hPCIS in tailor-made organoid media. These media consist of DMEM/F12 as a base medium, supplemented with organoid medium ingredients such as Wnt3a and R-spondin-1 (Table 1). We first assessed whether this base medium affected the general viability of hPCIS. Relative ATP/protein ratio was not affected by DMEM/F12 medium (Fig. S3A). The general morphology, visualized by HE staining, showed better preservation of the epithelium, but not crypts in DMEM/F12 cultured hPCIS compared to WME-cultured slices (Fig. S3B).

By carefully choosing the composition of these media, we tried to improve the stem cell viability with expansion medium (Emed) and enterocyte viability with differentiation medium (Dmed). The relative ATP/protein ratio of Dmed-cultured hPCIS incubated was similar to WME-cultured hPCIS, but the relative ATP/protein ratio of Emed-cultured hPCIS was significantly higher at 48 h of incubation (Fig. 3A). In contrast to WME-cultured hPCIS, Emed- and Dmed-cultured hPCIS did not show denudation of villi and less flattening and improved polarization of the epithelial cells. Moreover, crypts, where stem cells are located, are still present in Emed-, and Dmed- cultured hPCIS after 72 h of incubation. Thus, HE stainings of Emed-, and Dmed- cultured hPCIS showed a better morphology compared to slices incubated in WME (Fig. 3B and S4).

At the same time, after each incubation time-point, TUNEL staining showed fewer TUNEL positive cells in Emed, and Dmed-cultured hPCIS, whereas more KI67 positive cells were present in Emed-, and Dmed-cultured hPCIS compared to WME-cultured hPCIS (Fig. 3B and S4). This was also reflected in the gene expression of apoptosis and proliferation markers (Fig. 3C and S5). From the injury markers, only *HMOX1* was significantly upregulated in Dmed-cultured hPCIS compared to WME-cultured hPCIS after 48 h of incubation (Fig. 3C and S5). The expression of inflammation markers *IL-1B*, *IL-6* and, *TNF,* and fibrosis marker *PAI1* was significantly decreased in Emed-cultured hPCIS compared to WME-cultured hPCIS (Fig. 3C and S5). Taken together, hPCIS incubated in Emed or Dmed show improved general viability up to 72 h of incubation compared to WME cultured hPCIS.

### hPCIS epithelial layer can be rescued using specific organoid media

To investigate whether Emed or Dmed media could preserve the hPCIS epithelium better than WME, cell type-specific gene and protein expression and MIDA metabolism were assessed. Emed-cultured hPCIS showed a significant reduction of epithelial markers *EZR,* and *TJP-1,* while E-cadherin staining was better preserved compared to WME-cultured hPCIS. Dmed-cultured hPCIS showed similar gene expression of epithelial markers and E-cadherin staining was better maintained compared to WME-cultured hPCIS (Fig. 4A and B, S4 and S5). Emed-cultured hPCIS significantly increased expression of stem cell marker *LGR5* and maintained intracellular stem cell marker OLFM4 and proliferation marker KI67 stained cells located in the crypts (Figs. 3B, 4A and B, S4 and S5). Dmed-incubated hPCIS also showed increased positive staining for OLFM4 and KI67 compared to WME-incubated hPCIS, but not to the extent of hPCIS incubated in Emed medium (Figs. 3B, 4A and B, S3 and S5). In sections of Emed-, and Dmed-cultured hPCIS, the number of crypts present in slices cultured in Emed was clearly higher than in WME-cultured slices (Figs. 3B, 4B, and S3). The expression of Paneth cell markers was comparable between all media. Only *LYZ* expression, a Paneth cell marker, was significantly lower in Emed-cultured hPCIS incubated for 24 h and 72 h compared to WME-cultured hPCIS, although this was not reflected in the Lysozyme stainings (Fig. 4A and B, S4 and S5). Incubation in the Dmed medium resulted in better preservation of epithelial cells along the villi compared to incubation in WME or Emed media, as shown by a slight increase in epithelial, goblet cell, and enterocyte markers (Fig. 4A). No better preservation of goblet cells in either Emed- or Dmed- cultured hPCIS was visible on PAS stainings (Fig. 4B). Also, mucin quantification did not show significant differences in mucin production by Emed- or Dmed-cultured hPCIS compared to WME-cultured hPCIS (Fig. 4C). The expression of enterocyte-specific transporter *ABCB1* and enzymes *CYP3A4* and *CYP3A5* were higher in Dmed-cultured hPCIS and initially lower and at 72 h higher in Emed-cultured hPCIS compared to WME-cultured hPCIS (Fig. 4A). This was in line with the activity of these CYP3A4/5 enzymes, as measured by MIDA metabolite formation rates. Emed-cultured hPCIS showed lower (at 48 h significant) formation rates of 1-OH-MIDA compared to WME-cultured hPCIS and 4-OH-MIDA were not determined at any timepoint (Fig. 4D and E). The formation rate of 1-OH-MIDA was at least two times higher in Dmed-cultured hPCIS compared to WME-cultured hPCIS at all time points, which was significant at 48 h of incubation (Fig. 4D). The formation rate of 4-OH-MIDA was also higher after 48 h and 72 h of incubation (Fig. 4E). Remarkably, in hPCIS cultured in Dmed the *CYP3A4* and *CYP3A5* expression, as well as the MIDA metabolite formation, was not significantly different from the hPCIS directly after slicing, indicating that the drug-metabolizing capacity of enterocyte is preserved during the course of 72 h of culture in Dmed. Together, the results show that the supplements in Dmed steer towards better preserved and differentiated villi epithelium while the additives in Emed medium steer towards a better-preserved stem cell population, allowing preservation of functional hPCIS for up to 72 h.

Of note, gene expression of Wnt- and Notch target genes, *CCND1* and *HES1* were not increased in hPCIS cultured in either of the organoid media compared to hPCIS cultured in WME, Wnt target gene *LGR5*, however, was significantly increased in hPCIS cultured in Emed compared to hPCIS cultured in WME. *MYC* a target gene of Wnt signaling was significantly decreased in hPCIS cultured in Emed for 48 h compared to hPCIS cultured in WME (Fig. 4A and Supplementary Fig. S5).

## Discussion

PCIS prepared from the human intestine is a valuable model to study toxicology, pharmacokinetics, and transport of drugs in a relevant human setting (Li et al. 2016, 2017; Iswandana et al. 2018). Due to decreasing viability during culture, hPCIS can only reliably be cultured for a maximum of 24 h in WME. In this study, we aimed to improve the viability of hPCIS and to steer specifically towards proliferating stem cells and metabolically active enterocytes in hPCIS during culture by using tailor-made organoid media (Table 1). We confirm that, when instead of the standard WME culture medium, organoid media are used, hPCIS viability, as characterized by polarized epithelium, proliferating crypt cells, and the activity of CYP enzymes can be maintained for up to 72 h of incubation.

Directly after the slicing procedure, hPCIS epithelium and crypt morphology and metabolic function of CYP3A4 and CYP3A5 are intact, making hPCIS suitable for short-time—hours—experiments. However, in line with previous publications (Bigaeva et al. 2019a; Biel et al. 2020), we show that hPCIS viability and cellular heterogeneity/survival deteriorated during the course of 72 h of incubation in WME (Figs. 1 and 2). Especially, a rapid decline of stem cells was determined, as shown by a decline in *LGR5* and *OLFM* mRNA expression*,* as well as a decrease in positive OLFM4 stain during 72 h of hPCIS incubation (Fig. 2A and B). Moreover, enterocyte functionality also declines during incubation, as shown by a decline in MIDA metabolite formation after MIDA exposure (Fig. 2D and E). Moreover, Martinec et al., showed a gradual decline in protein expression of ABCB1 during hPCIS incubation (Martinec et al. 2021).

The decline in cellular diversity and viability has a similar pattern in hPCIS compared to mouse and rat PCIS (Biel et al. 2020). While the majority of the cell type markers gradually decreased during incubation, two markers for goblet cells and stem cells did not change. Mucin excreted in the culture medium did not change over time; this is in contrast to the upregulation of *MUC2* as well as the decline of the amount of mucin-containing goblet cells determined by the PAS staining. This is in line with our previous study in mouse and rat PCIS and can be caused by the fact that using ELLA, not only goblet cell-specific mucins are measured, but also mucins excreted by, among others, enterocytes (Lee et al. 2013; Pelaseyed et al. 2014). Moreover, it is possible that goblet cells release their mucin during incubation and therefore are ‘empty’, resulting in the absence of mucin-containing goblet cells that are not visible on PAS staining but express *MUC2* and secrete mucin in the culture medium (Fig. 2).

Interestingly, while the stem cell markers *LGR5* and *OLFM4* decreased during incubation, *BMI1* expression increased during incubation of hPCIS in WME. *LGR5* and *OLFM4* are markers for actively cycling intestinal stem cells (crypt-based columnar cells) (van der Flier et al. 2009; Sato and Clevers 2013). In contrast, *BMI1* is a marker for quiescent (slowly cycling) intestinal stem cells (Takeda et al. 2011; Leushacke and Barker 2014). In intestinal injury models (radiation, ischemia), it has been shown that actively cycling (LGR5^+^) stem cells die fast (within hours) upon injury, while quiescent stem cells are more resistant and eventually start regenerating the intestinal tissue (Takeda et al. 2011; Roth et al. 2012; Gonzalez et al. 2019). Moreover, it has been shown that the quiescent stem cells express *CCND1* post-injury, indicating proliferation. We also observed an increase (ns) in *CCND1* during hPCIS incubation (Fig. 2B). Similar to other injury models, the quiescent stem cells in hPCIS may be activated due to the injury induced by the ischemia time and slicing procedure, however, this needs to be further elucidated.

Paneth cells support intestinal stem cells, among others, via Notch signaling (Kriz and Korinek 2018). mRNA expression of Paneth cell markers *LYZ* and *DEF5A* decreases during incubation of hPCIS in WME. Previously, we also showed that Paneth cells are lost during the culture of mouse and rat PCIS (Biel et al. 2020). Interestingly, Notch downstream target *HES1* (together with *MYC* and *CCND1*) mRNA expression increased during incubation of hPCIS. One reason for upregulated *HES1* might be the inflammatory environment of all epithelial cells in the hPCIS during culture. Similar expression patterns are found in the inflamed intestine of mice, and ulcerative colitis patients (Okamoto et al. 2009; Zheng et al. 2011; Shimizu et al. 2014). It was shown that *HES1* expression is increased in the inflamed intestinal epithelium compared to the normal epithelium.

hPCIS have been used for short-term pharmacokinetics (drug metabolism and transport) and toxicology studies of drugs (Niu et al. 2013; Li et al. 2016; Iswandana et al. 2018; Martinec et al. 2021). For long-term pharmacokinetic and toxicology studies, viable and metabolic active enterocytes are needed. In this study, we aimed to improve hPCIS viability and cellular heterogeneity by supporting stem cell maintenance, proliferation, and/or enterocyte differentiation by culturing in organoid media.

In vivo, intestinal crypt-based stem cells take care of the renewal and maintenance of the intestinal epithelium. A few pathways are important in stem cell maintenance and crypt proliferation, for example, the Wnt- and notch-signaling pathways. The canonical Wnt-pathway is activated via the binding of Wnt3a to the LGR5 receptor. This leads to the stabilization of β-catenin, which can then translocate to the nucleus. In the nucleus, β-catenin interacts with TCF/LEF and leads to transcription of target genes such as *c-MYC, CCND1,* and *AXIN2* (Leushacke and Barker 2014)*.* R-Spondin-1 is an agonist of this Wnt-pathway, and together they drive stem cell faith and proliferation (Holmberg et al. 2017). On the other hand, Notch-signaling is essential to maintain the undifferentiated state of stem cells. Paneth cells, located next to the intestinal stem cells support stem cells via Notch signaling. Paneth cells express DII1 and DII4 on the cell membrane, these proteins bind to Notch. Subsequently, Notch interacts with a nuclear effector (RBP-J) and thereby suppresses the gene expression of genes involved in secretory lineage differentiation (Sato and Clevers 2013). In vivo, agonists, and antagonists of the above-mentioned pathways are present in a gradient from the crypt to villus tip to enhance stemness and proliferation in the crypt or differentiation towards the villus tip (Sato and Clevers 2013; Leushacke and Barker 2014). Ex vivo in PCIS, this gradient might be faded out due to dilution in the culture medium. Moreover, the cells producing the above-mentioned molecules might not be functional anymore due to injury, and therefore, among other reasons, stem cell maintenance and proliferation might be interrupted.

Previously, we have shown in mouse and rat PCIS, that growth factors of the stem cell niche can help maintain and improve PCIS viability and morphology (Bigaeva et al. 2019a; Biel et al. 2020). In these studies, we recommended using complete organoid media, including compounds, such as CHIR99021, Y27632, or Valproic acid (Sato et al. 2011; Leushacke and Barker 2014). In Emed and Dmed, Stem cell maintenance and proliferation were promoted by Wnt-signaling agonists (Wnt3a, R-spondin-1), and differentiation was prevented using an antagonist of BMP-signaling (DMH1). R-spondin-1 is an agonist of Wnt-signaling, which is necessary for stem cell maintenance and proliferation (Holmberg et al. 2017). DMH-1 is a small molecule inhibitor of BMP, replacing the function of Noggin, which stimulates crypt formation (Holmberg et al. 2017). The Emed medium, in addition, also contained CHIR99021, Valproic acid, and, Y27632, which stimulate Wnt-signaling, Notch signaling, and inhibit caspase-3, respectively (Holmberg et al. 2017). This should steer stem cell maintenance, and block differentiation and cell death (Yin et al. 2014; Tong et al. 2018). Upon culture of hPCIS in Emed or Dmed, Wnt and Notch agonist activity of Wnt3a, R-spondin-1, CHIR99021, and Valproic acid was only confirmed by increased gene expression of *LGR5*. Other downstream Wnt targets (*MYC* and *CCND1*) did not increase in hPCIS incubated in Emed or Dmed. Notch downstream target *HES1* also did not change upon culture in Emed and Dmed compared to WME. These unexpected results might be related to the complex cellular composition of hPCIS and/or timing of the gene expression analysis. Other cell types may mask changes in gene expression in the small population of intestinal stem cells. On the other hand, as mentioned above, stem cell and crypt signaling is disturbed in inflammatory conditions. Emed contained Y27632, this compound prevents apoptosis via inhibition of caspase-3 (Sato et al. 2011). Indeed, we found a decrease in *TP53* mRNA expression and TUNEL positive staining in Emed-cultured hPCIS compared to WME and Dmed-cultured hPCIS. Next to the anti-apoptosis function of Y27632, it also acts as a ROCK-inhibitor and thereby inhibiting inflammation and fibrogenesis. This is also seen in the mRNA expression data of *IL-6*, *IL-1B*, *TNF*, and *PAI1*.

Although downstream targets of Wnt and Notch did not show the expected activation of these pathways, incubation of hPCIS in organoid media resulted in the maintenance of the proliferating stem cells, as shown by increased expression of *LGR5* and *OLFM4* and *MKI67*. Moreover, hPCIS morphology was improved by Emed-cultured hPCIS as it showed better preservation of both epithelium as well as crypts compared to WME-cultured hPCIS, while in Dmed-cultured hPCIS only epithelial cells were better maintained. Dmed did not contain Wnt and Notch agonists, CHIR99021, and Valproic acid, allowing cells to further mature towards enterocytes (Brunck and Nielsen 2014). Indeed, epithelial cells were better maintained in Dmed-cultured hPCIS compared to Emed-cultured hPCIS, as shown by gene expression analysis of epithelial markers and E-cadherin protein expression. More importantly, Dmed-cultured hPCIS had a higher expression of *ABCB1*, *CYP3A4*, and *CYP3A5*, indicating better maintenance of enterocytes and possible increased differentiation of stem cells towards enterocytes. The activity of important drug-metabolizing enzymes CYP3A4 and CYP3A5 was improved in Dmed-cultured hPCIS compared to WME-cultured hPCIS. Even after 72-h incubation, the CYP3A4 and CYP3A5 activity in Dmed-cultured hPCIS was similar to hPCIS directly after the slicing procedure, making them suitable for long-term pharmacokinetic and toxicology studies. To further validate the use of this culture medium and its components, in-depth characterization of multiple drug-transporters and drug-metabolizing enzymes is needed.

The use of human-derived tissue, including the hPCIS, is characterized by high interindividual variability. This is the result of patient characteristics, such as genetics, underlying disease, and medication. Especially when studying the metabolism and transport of drugs, this might be a challenge, since it is known that metabolizing enzymes and transporters are highly affected by these factors (Hohmann et al. 2016). Also in our data, we observed interindividual differences in the expression of *CYP3A4* and *CYP3A5* (Maximum fold change difference of 12 and 3.7 respectively). Moreover, the extent of the beneficial effect of the two different organoid media compared to the WME also differed per individual donor. When making use of these individual differences, hPCIS might be an interesting tool in the context of personalized medicine.

Using two organoid-tailored culture media, we have demonstrated that it is possible to extend the hPCIS viability and improve the cellular heterogeneity, morphology, and metabolic capacity of hPCIS for up to 72 h of incubation. With the obtained results of hPCIS cultured in a tailor-made organoid medium, this model can serve as a clinically relevant, animal-free model for pharmacokinetic, metabolism, and toxicity studies in the intestine.

## Supplementary Information

Below is the link to the electronic supplementary material.

Supplementary file1 (PDF 1612 KB)

## References

[CR1] Biel C, Bigaeva E, Hesse M (2020). Survival and cellular heterogeneity of epithelium in cultured mouse and rat precision-cut intestinal slices. Toxicol Vitr.

[CR2] Bigaeva E, Bomers JJM, Biel C (2019). Growth factors of stem cell niche extend the life-span of precision-cut intestinal slices in culture: a proof-of-concept study. Toxicol Vitr.

[CR3] Bigaeva E, Gore E, Simon E (2019). Transcriptomic characterization of culture-associated changes in murine and human precision-cut tissue slices. Arch Toxicol.

[CR4] Brunck MEG, Nielsen LK (2014). Concise review: next-generation cell therapies to prevent infections in neutropenic patients. Stem Cells Transl Med.

[CR8] de Graaf IAM, Olinga P, de Jager MH (2010). Preparation and incubation of precision-cut liver and intestinal slices for application in drug metabolism and toxicity studies. Nat Protoc.

[CR5] Dedhia PH, Bertaux-Skeirik N, Zavros Y, Spence JR (2016). Organoid models of human gastrointestinal development and disease. Gastroenterology.

[CR7] Gonzalez LM, Stewart AS, Freund J (2019). Preservation of reserve intestinal epithelial stem cells following severe ischemic injury. Am J Physiol Liver Physiol.

[CR9] Hohmann N, Haefeli WE, Mikus G (2016). CYP3A activity: towards dose adaptation to the individual. Expert Opin Drug Metab Toxicol.

[CR10] Holmberg FE, Seidelin JB, Yin X (2017). Culturing human intestinal stem cells for regenerative applications in the treatment of inflammatory bowel disease. EMBO Mol Med.

[CR11] Iswandana R, Irianti MI, Oosterhuis D (2018). Regional differences in human intestinal drug metabolism. Drug Metab Dispos.

[CR13] Khan AA, Chow ECY, van Loenen-Weemaes AMA (2009). Comparison of effects of VDR versus PXR, FXR and GR ligands on the regulation of CYP3A isozymes in rat and human intestine and liver. Eur J Pharm Sci.

[CR14] Khan AA, Chow ECY, Porte RJ (2009). Expression and regulation of the bile acid transporter, OST α -OST β in rat and human intestine and liver. Biopharm Drug Dispos.

[CR15] Kozuka K, He Y, Koo-McCoy S (2017). Development and characterization of a human and mouse intestinal epithelial cell monolayer platform. Stem Cell Reports.

[CR16] Kriz V, Korinek V (2018). Wnt, RSPO and hippo signalling in the intestine and intestinal stem cells. Genes.

[CR17] Lee C-SS, Muthusamy A, Abdul-Rahman PS (2013). An improved lectin-based method for the detection of mucin-type O-glycans in biological samples. Analyst.

[CR18] Leushacke M, Barker N (2014). Ex vivo culture of the intestinal epithelium: strategies and applications. Gut.

[CR19] Li M, de Graaf IAM, de Jager MH, Groothuis GMM (2017). P-gp activity and inhibition in the different regions of human intestine ex vivo. Biopharm Drug Dispos.

[CR20] Li M, de Graaf IAM, Groothuis GMM (2016). Precision-cut intestinal slices: alternative model for drug transport, metabolism, and toxicology research. Expert Opin Drug Metab Toxicol.

[CR21] M.M. Groothuis G, A.M. de Graaf I,  (2012). Precision-cut intestinal slices as in vitro tool for studies on drug metabolism. Curr Drug Metab.

[CR22] Martignoni M, Groothuis G, de Kanter R (2006). Comparison of mouse and rat cytochrome P450-mediated metabolism in liver and intestine. Drug Metab Dispos.

[CR23] Martignoni M, Groothuis GMM, de Kanter R (2006). Species differences between mouse, rat, dog, monkey and human CYP-mediated drug metabolism, inhibition and induction. Expert Opin Drug Metab Toxicol.

[CR24] Martinec O, Biel C, de Graaf IAM (2021). Rifampicin induces gene, protein, and activity of p-glycoprotein (ABCB1) in human precision-cut intestinal slices. Front Pharmacol.

[CR25] Niu X, de Graaf IAM, Groothuis GMM (2013). Evaluation of the intestinal toxicity and transport of xenobiotics utilizing precision-cut slices. Xenobiotica.

[CR26] Okamoto R, Tsuchiya K, Nemoto Y (2009). Requirement of notch activation during regeneration of the intestinal epithelia. Am J Physiol Gastrointest Liver Physiol.

[CR27] Pelaseyed T, Bergström JH, Gustafsson JK (2014). The mucus and mucins of the goblet cells and enterocytes provide the first defense line of the gastrointestinal tract and interact with the immune system. Immunol Rev.

[CR28] Pham BT, van Haaften WT, Oosterhuis D (2015). Precision-cut rat, mouse, and human intestinal slices as novel models for the early-onset of intestinal fibrosis. Physiol Rep.

[CR29] Roth S, Franken P, Sacchetti A (2012). Paneth cells in intestinal homeostasis and tissue injury. PLoS ONE.

[CR30] Sato T, Clevers H (2013). Growing self-organizing mini-guts from a single intestinal stem cell: mechanism and applications. Science.

[CR31] Sato T, Stange DE, Ferrante M (2011). Long-term expansion of epithelial organoids from human colon, adenoma, adenocarcinoma, and Barrett’s epithelium. Gastroenterology.

[CR32] Scarpignato C, Bjarnason I (2019). Drug-induced small bowel injury: a challenging and often forgotten clinical condition. Curr Gastroenterol Rep.

[CR33] Shanks N, Greek R, Greek J (2009). Are animal models predictive for humans? Philos ethics. Humanit Med.

[CR34] Shimizu H, Okamoto R, Ito G (2014). Distinct expression patterns of Notch ligands, Dll1 and Dll4, in normal and inflamed mice intestine. PeerJ.

[CR35] Takeda N, Jain R, LeBoeuf MR (2011). Interconversion between intestinal stem cell populations in distinct niches. Science.

[CR36] Tong Z, Martyn K, Yang A (2018). Towards a defined ECM and small molecule based monolayer culture system for the expansion of mouse and human intestinal stem cells. Biomaterials.

[CR12] van de Kerkhof EG, de Graaf IAM, Ungell A-LB, Groothuis GMM (2008). Induction of metabolism and transport in human intestine: validation of precision-cut slices as a tool to study induction of drug metabolism in human intestine in vitro. Drug Metab Dispos.

[CR6] van der Flier LG, Haegebarth A, Stange DE (2009). OLFM4 Is a robust marker for stem cells in human intestine and marks a subset of colorectal cancer cells. Gastroenterology.

[CR37] Wessels AMA, Bolhuis MS, Bult W (2021). A fast and simple method for the simultaneous analysis of midazolam, 1-hydroxymidazolam, 4-hydroxymidazolam and 1-hydroxymidazolam glucuronide in human serum, plasma and urine. J Chromatogr B.

[CR38] Yin X, Farin HF, van Es JH (2014). Niche-independent high-purity cultures of Lgr5+ intestinal stem cells and their progeny. Nat Methods.

[CR39] Zheng X, Tsuchiya K, Okamoto R (2011). Suppression of hath1 gene expression directly regulated by hes1 via notch signaling is associated with goblet cell depletion in ulcerative colitis. Inflamm Bowel Dis.

