# Supplementary material for: Extending the viability of human precision-cut intestinal slice model for drug metabolism studies
Source: Arch Toxicol. 2022 Apr 15;96(6):1815–27. doi: 10.1007/s00204-022-03295-1 (PMC9095520; doi:10.1007/s00204-022-03295-1)
Supplement: Supplementary file 1 — Supplementary file1 (PDF 1612 KB) [file 204_2022_3295_MOESM1_ESM.pdf]

Table S1 *Primer sequences*

Gene	Forward sequence	Reverse sequence
RPLP0	CAGATTGGCTACCCAACTGTT	GGAAGGTGTAATCCGTCTCCAC
LGR5	CACCTCCTACCTAGACCTCAGT	CGCAAGACGTAACTCCTCCAG
OLFM4	ACTGTCCGAATTGACATCATGG	TTCTGAGCTTCCACCAAAACTC
BMI1	GCTGCCAATGGCTCTAATGAA	TGCTGGGCATCGTAAGTATCTT
EZR	ATGCCCCACGTCTGAGAATC	TCCTGCGGCGCATATACAAC
TJP-1	ACCAGTAAGTCGTCCTGATCC	TCGGCCAAATCTTCTCACTCC
CDH1	CGAGAGCTACACGTTCACGG	GGGTGTCGAGGGAAAAATAGG
CDX2	GACGTGAGCATGTACCCTAGC	GCGTAGCCATTCCAGTCCT
LYZ	TCAATAGCCGCTACTGGTGTA	ATCACGGACAACCCTCTTTGC
DEF5A	AGACAACCAGGACCTTGCTAT	GGAGAGGGACTCACGGGTAG
MUC2	GAGGGCAGAACCCGAAACC	GGCGAAGTTGTAGTCGCAGAG
MYC	GGCTCCTGGCAAAAGGTCA	CTGCGTAGTTGTGCTGATGT
CCND1	GCTGCGAAGTGGAAACCATC	CCTCCTTCTGCACACATTTGAA
HES1	CCTGTCATCCCCGTCTACAC	CACATGGAGTCCGCCGTAA
CYP3A4	GCCTGGTGCTCCTCTATCTA	GGCTGTTGACCATCATAAAAG
CYP3A5	ATCGAAGGTCTTTAGGCCCAG	CTTCCCGCCTCAAGTTTCTC
ABCB1	GGGATGGTCAGTGTTGATGGA	GCTATCGTGGTGGCAAACAATA
HSPA4L	CGGCTTTCTCAACTGCTACAT	ACCTGTCGCTGTACTCATTGG
HMOX1	AAGACTGCGTTCCTGCTCAAC	AAAGCCCTACAGCAACTGTCG
IL6	ACTCACCTCTTCAGAACGAATTG	CCATCTTTGGAAGGTTCAGGTTG
IL1B	ATGATGGCTTATTACAGTGGCAA	GTCGGAGATTCGTAGCTGGA
TNF	GAGGCCAAGCCCTGGTATG	CGGGCCGATTGATCTCAGC
FN1	CGGTGGCTGTCAGTCAAAG	AAACCTCGGCTTCCTCCATAA
PAI1	ACCGCAACGTGGTTTTCTCA	TTGAATCCCATAGCTGCTTGAAT
ACTA2	AAAAGACAGCTACGTGGGTGA	GCCATGTTCTATCGGGTACTTC
TP53	ACAGCTTTGAGGTGCGTGTTT	CCCTTTCTTGCGGAGATTCTCT
BAX	CCCGAGAGGTCTTTTTCCGAG	CCAGCCCATGATGGTTCTGAT
MKI67	ACGCCTGGTTACTATCAAAAGG	CAGACCCATTTACTTGTGTTGGA

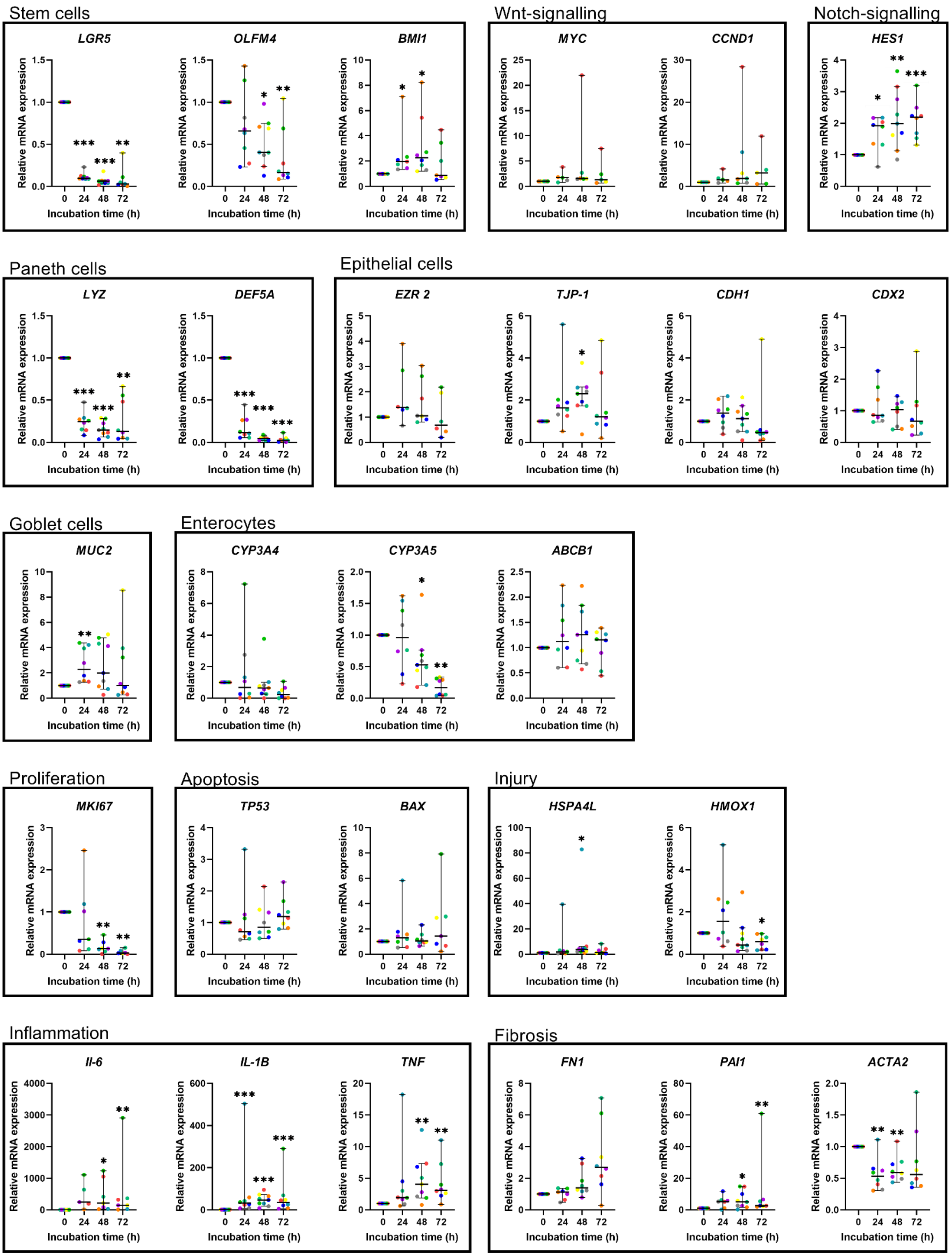

Fig. S1 *Gene expression analysis of WME-cultured hPCIS cultured for up to 72 h. Dots represent relative gene expression of hPCIS from individual donors the horizontal lines indicate mean ± SEM. REML followed by Dunnett's post-hoc test was performed to calculate statistical differences. *p<0.05, **p<0.01, ***p<0.005.*

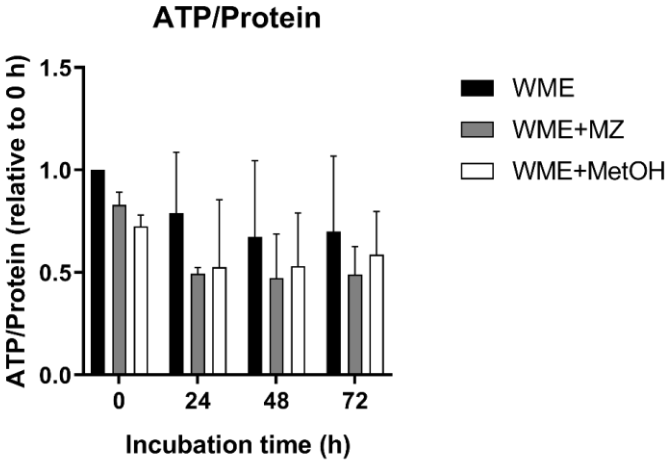

Fig. S2 *Relative ATP/protein ratios hPCIS incubated in WME with and without Midazolam (MZ) and/or Methanol (MetOH). Bars indicate mean + SEM, n=3.*

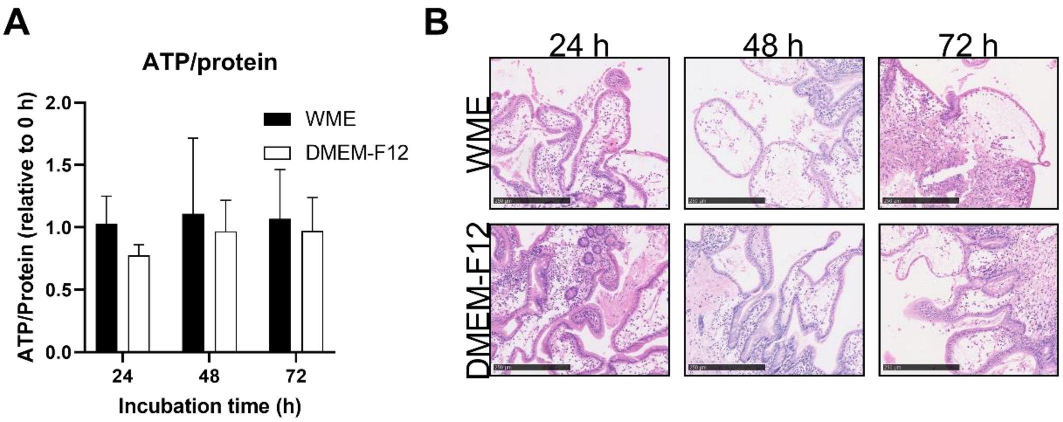

Fig. S3 *General viability of hPCIS cultured in WME or DMEM/F12 media. hPCIS were incubated in WME or DMEM/F12 for up to 72 h. (A) relative ATP/protein ratios were determined. Bars indicate mean + SEM. hPCIS were stained with HE. Images were taken at 40x magnification, bars are 250 µm.*

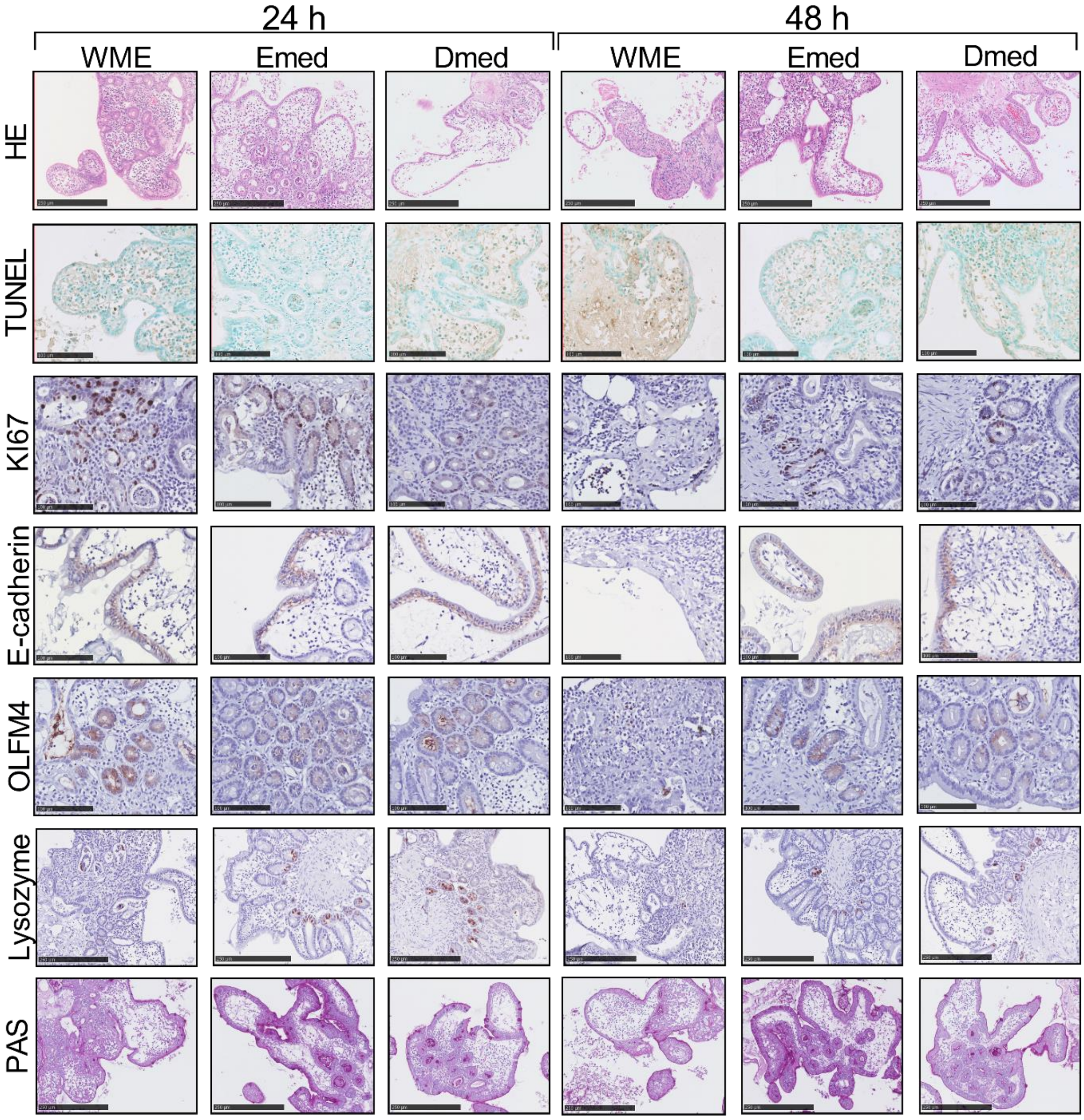

Fig. S4 *Representative photomicrographs of hPCIS incubated for 24 or 48 h in WME, Emed, or Dmed medium stained with HE, PAS, TUNEL or against KI67, E-cadherin, OLFM4, and Lysozyme. Photos were taken at 40X magnification, bars indicate 100 µm for KI67, E-cadherin, and OLFM4 stained sections, and 250 µm for HE, TUNEL, Lysozyme, and PAS stained sections.*

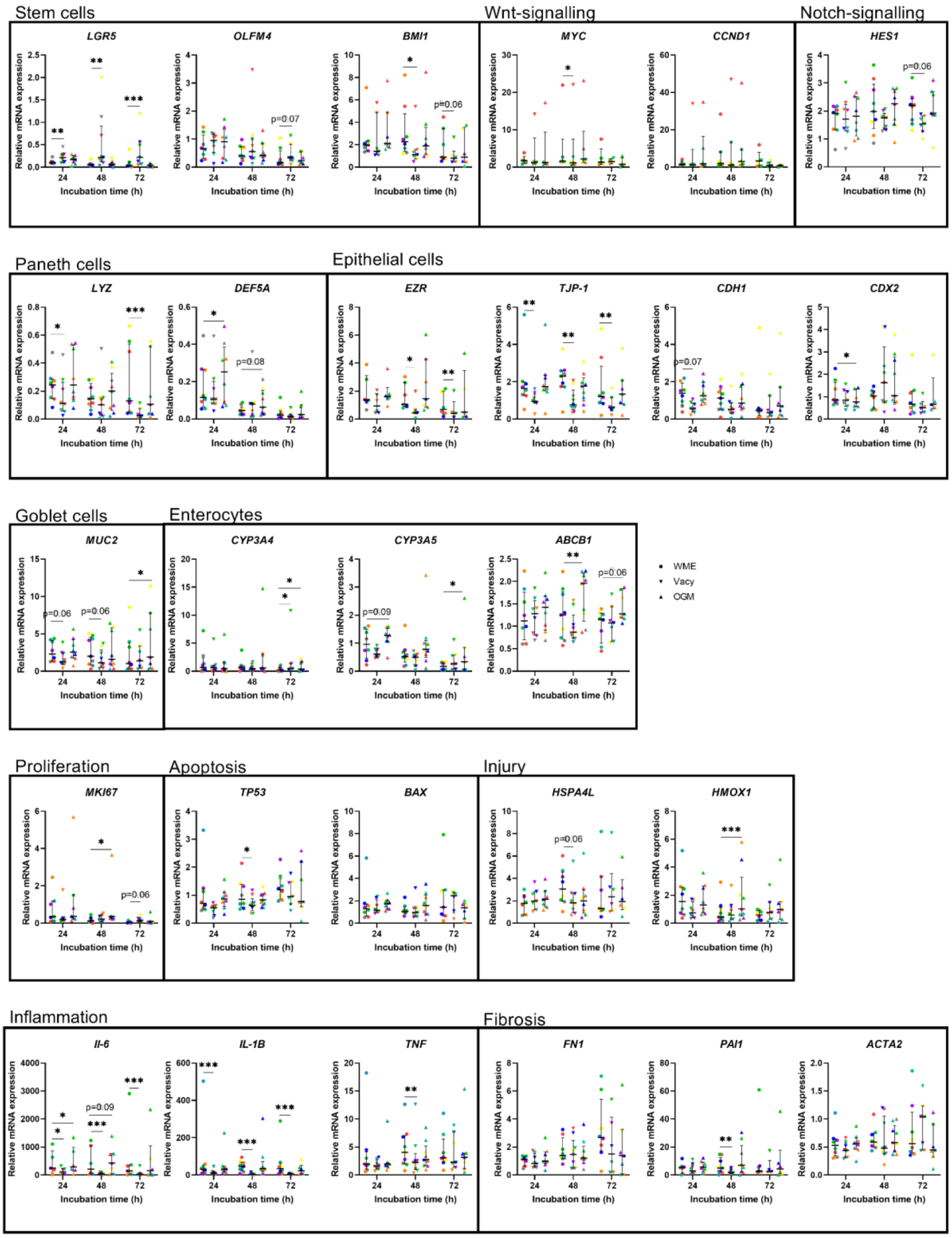

Fig. S5 *Gene expression analysis of hPCIS cultured in WME or DMEM-F12 for up to 72 h. Dots represent relative gene expression of hPCIS from individual donors the horizontal lines indicate mean ± SEM. Two-way ANOVA or REML followed by Dunnett's post-hoc test was performed to calculate statistical differences. *p<0.05, **p<0.01, ***p<0.005.*
